# Data on bond strength of methyl methacrylate-based resin cement to dental restorative materials

**DOI:** 10.1016/j.dib.2020.106426

**Published:** 2020-10-20

**Authors:** Hiroshi Ikeda, Pirat Karntiang, Yuki Nagamatsu, Hiroshi Shimizu

**Affiliations:** a Division of Biomaterials, Department of Oral Functions, Kyushu Dental University, Fukuoka 803-8580, Japan; b Division of Operative Dentistry, College of Dental Medicine, Rangsit University, Pathum Thani 12000, Thailand

**Keywords:** Bond strength, Adhesive, Primer, Dental alloy, Dental ceramic, Porcelain, Zirconia, 4-META/MMA-TBB resin cement

## Abstract

A methyl methacrylate (MMA) -based resin cement is one of the popular luting agents to bond a dental restorative material in dental treatments. Bond strength of the MMA-based resin cement with adhesive primer to each restorative material is important for clinical success in prosthodontic treatments without debonding or fracture failures of the restoration such as a dental crown and post. However, open data on the bonding properties of combined use of the MMA-based resin cement and appropriate primers is limited. This article provides data on the bond strength and fracture mode of the 4-META/MMA-TBB resin cement (MMA-based resin cement) to the restorative materials (silver alloy, gold alloy, feldspathic porcelain, and zirconia) with four types of primer. Each restorative material was applied with the adhesive primer and bonded with the MMA-based resin cement. The cement-bonded samples were subjected to a thermocycling in which the materials were immersed alternately in water baths at 5 °C and 55 °C for 10,000 cycles. The bond strength between the resin cement and each restorative material was measured by means of a conventional tensile bond strength test. The fracture modes of the examined samples were observed and determined. The measured tensile bond strengths were statistically analysed using one-way analysis of variance (ANOVA), followed by Tukey's multiple comparison test.

## Specifications Table

**Table:** 

Subject	Biomaterials
Specific subject area	Dental materials
Type of data	Table Figure
How data were acquired	Tensile bond strength was measured using a universal testing machine. Data was analysed using a statistical software, EZR (Saitama Medical Center, Jichi Medical University).
Data format	Raw Analyzed
Parameters for data collection	Combination use of four types of commercial dental restorative materials (silver alloy, gold alloy, feldspathic porcelain, zirconia) and four types of a commercial adhesive primers (V-PRIMER, Super-Bond Universal Ceramic Primer, Porcelain Liner M, M&C PRIMER, and no primer).
Description of data collection	Each dental indirect restorative material bonded to the MMA-based resin cement with the tested primers. After a thermocycling, the tensile bond strength between the dental material and the resin cement was measured by a conventional tensile bond strength test. The fracture modes of the tested samples were determined by an observation.
Data source location	Division of Biomaterials, Department of Oral Functions, Kyushu Dental University, 2-6-1 Manazuru, Kokura-kitaku, Kitakyushu, Fukuoka 803-8580, Japan
Data accessibility	All data herein and supplementary files are available within this article.
Related research article	None.

## Value of the Data

• The data, bond strength between the dental restorative material and the MMA-based resin cement, is important for a long-term success in prosthodontic treatments with avoiding a debonding failure.
• The data can help dentists to choose appropriate primer when the dental restorative material is to be bonded to tooth using the MMA-based resin cement in prosthodontic treatments.
• The data can be compared with that on the other collected data for various kinds of dental restorative materials, including alloys, ceramics, and composites.
• The data can help to obtain bonding properties of the MMA-based resin cement with the adhesive primers to dental restorative materials.

## 1 Data Description

**Fig. 1:**
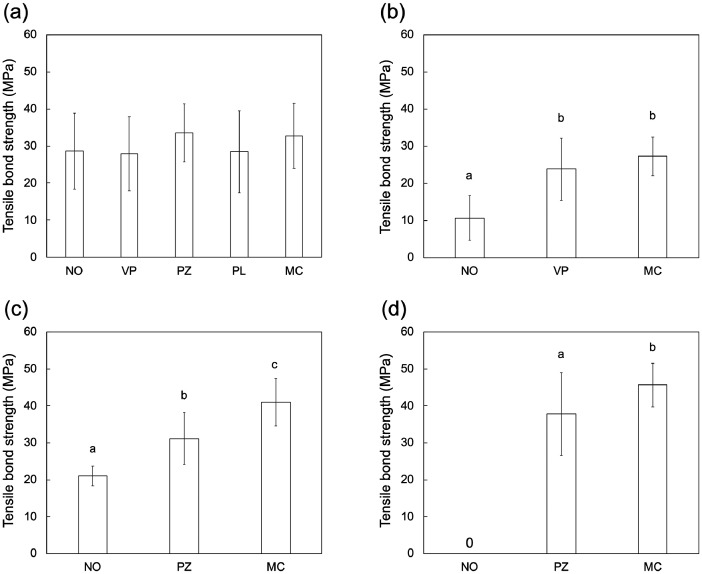
Tensile bond strengths between the MMA-based resin cement with the adhesive primers and the restorative materials: (a) silver alloy, (b) gold alloy, (c) zirconia, and (d) feldspathic porcelain. The abbreviations of the primers; NO: no primer use, VP: V-PRIMER, PZ: Super-Bond Universal Ceramic Primer, PL: Porcelain Liner M, MC: M&C PRIMER. The letters indicate a significant difference between the groups (p < 0.05, Tukey test, n = 11).

The datasets provide information on bonding properties (bond strength and fracture mode) of the dental restorative materials to the 4-META/MMA-TBB resin cement (MMA-based resin cement) using the adhesive primers. Table 1 gives detail of the commercial MMA-based resin cement used in this experiment. Table 2 depicts the adhesive primers that are recommended by the company to apply to each restorative material; V-PRIMER for noble metal alloys (e.g. gold alloys), Super-Bond Universal Ceramic Primer for both glass-ceramics (e.g. feldspathic porcelain and lithium disilicate glass) and polycrystalline-ceramics (e.g. zirconia), Porcelain Liner M for both the glass-ceramics and polycrystalline-ceramics, M&C PRIMER for any materials (e.g. noble metal alloys, base metal alloys, glass-ceramics, polycrystalline-ceramics). Table 3 lists the restorative materials including two types of dental alloys (silver and gold alloys) and two types of dental ceramics (feldspathic porcelain and zirconia). Fig. 1 shows the bond strength between each restorative material and the MMA-based resin cement with tested primers after thermocycling. The tensile bond strengths of the groups with different primer used were statistically compared in each restorative material using one-way analysis of variance (ANOVA) followed by Tukey's multiple comparison tests; the results are indicated in Fig. 1. Table 4 lists fracture modes of examined sample after the tensile bond strength test. The raw data of the tensile bond strength and fracture mode includes supplementary data files.

**Table 1:** The 4-META/MMA-TBB resin cement used in this experiment.

Trade name	Manufacturer	Constituent	Lot number	Composition	Ref.
		Polymer powder clear	SM11	PMMA	
Super-Bond	Sun Medical Co., Ltd	Quick monomer liquid	SE2	4-META, MMA	[1]
		Catalyst V	SM12	TBB	

PMMA: poly(methyl methacrylate), 4-META: 4-methacryloxyethyl trimellitate anhydride, MMA: methyl methacrylate, TBB: Tri-n-butylborane.

**Table 2:** The adhesive primers used in this experiment.

Trade name	Manufacturer	Lot number	Composition	Ref.
V-PRIMER	Sun Medical Co., Ltd	SS1	VTD, acetone	[2]
Super-Bond Universal Ceramic Primer*	Sun Medical Co., Ltd	RS12 (liquid A) SG12 (liquid B)	Liquid A: MDP, MMA Liquid B: γ-MPTS, MMA	[3]
Porcelain Liner M	Sun Medical Co., Ltd	MM1F (liquid A) SG12 (liquid B)	Liquid A: 4-META, MMA Liquid B: γ-MPTS, MMA	[4]
M&C PRIMER	Sun Medical Co., Ltd	18160 (liquid A) SG12 (liquid B)	Liquid A: MDP, VTD, MMA, acetone Liquid B: γ-MPTS, MMA	[5]

VTD: 6-(4-vinylbenzyl-n-propyl)amino-1,3,5-triazine-2,4-dithione, MDP: 10-methacryloyloxydecyl dihydrogen phosphate, MMA: methyl methacrylate, γ-MPTS: 3-methacryloxypropyl trimethoxy silane.

⁎ Trade name in Japan: Super-Bond PZ Primer.

**Table 3:** The restorative materials used in this experiment.

Material type	Trade name	Manufacturer	Lot number	Composition	Ref.
Silver alloy	Miro 3	GC Corp.	1302121	Ag 77, Sn 18, Zn 5 (mass%)	[6]
Gold alloy	Casting Gold M.C. type IV	GC Corp.	1011071	Au 70, Pt 2, Pd 3, Ag 8, Cu 16, Zn and Ir 1 (mass%)	[7]
Feldspathic porcelain	Super Porcelain AAA	Kuraray Noritake Dental Inc.	DNQVH	Potassium-aluminosilicate glass, others	[8]
Zirconia	SZY-H	SHINAGAWA FINE CERAMICS CO., LTD.	20040331	ZrO_2_ 93.9, Y_2_O_3_ 5.4, other 0.7 (mass%)	[9]

**Table 4:** Fracture modes of the debonded samples after the tensile bond strength test; (a) silver alloy, (b) gold alloy, (c) zirconia, and (d) feldspathic porcelain. The abbreviations of the primers; NO: no primer use, VP: V-PRIMER, PZ: Super-Bond Universal Ceramic Primer, PL: Porcelain Liner M, MC: M&C PRIMER.

Material	Primer	Number of failure modeD / A / C / F
	NO	0 / 1 / 10 / 0
	VP	0 / 1 / 10 / 0
Silver alloy	PZ	0 / 2 / 9 / 0
	PL	0 / 4 / 7 / 0
	MC	0 / 1 / 10 / 0
	NO	0 / 11 / 0 / 0
Gold alloy	VP	0 / 9 / 2 / 0
	MC	0 / 8 / 3 / 0
	NO	0 / 10 / 1 / 0
Zirconia	PZ	0 / 0 / 11 / 0
	MC	0 / 0 / 11 / 0
	NO	11 / 0 / 0 / 0
Porcelain	PZ	0 / 4 / 6 / 1
	MC	0 / 3 / 7 / 1

Fracture modes; debonding before the tensile bond strength test (D), cohesive failure in the cement (C), mixture of adhesive failure at the cement–restorative material interface and cohesive failure (A), failure of the restorative materials (F).

## 2 Materials and Methods

### 2.1 Sample preparation

The silver and gold alloy ingots were formed into 10 × 10 × 2.7 mm-plate and 10 × 10 × 4.5 mm-plate, respectively, by a conventional casting method according to the manufacturer's instruction. The sintered zirconia block was cut into 10 × 10 × 5 mm-plate. The feldspathic porcelain was formed into a disc with 16 mm in diameter, 10 mm in thickness via a sintering process according to the manufacturer's instruction. Each material was polished using an emery papers up to #2,000 and cleaned by ultrasonic water treatment for 10 min, subsequently dried in air. A masking tape with 4.8-mm-diameter hole was attached to the polished surface to standardize bonding area. The material surface was applied with the adhesive primer and then dried by air-blowing. The primed surface was cemented with the MMA-based resin cement and bonded to a stainless-steel rod. Subsequently the sample was kept in room temperature (25 ± 3 °C) for 30 min to cure the MMA-based resin cement. The cement-bonded sample was subjected to thermocycling process for 10,000 cycles which was conducted by alternating immersion in water baths at 5 °C and 55 °C with a 20 s dwell time for each bath. The resultant samples were used for the tensile bond strength test (n = 11).

### 2.2 Tensile bond strength test

Bond strength between the resin cement and the restorative material was measured by means of a conventional tensile bond strength test using universal testing machine (AGS-H, Shimadzu Corp., Japan) with a crosshead speed of 2 mm/min. The maximum load was recorded when the resin cement debonded from the material surface. The bond strength was calculated by dividing the maximum load by the bonding area.

### 2.3 Determination of fracture mode

The debonded surface of the material after the tensile bond strength test was observed by optical microscope and naked eyes to determine a fracture mode; debonding before the tensile bond strength test (D), cohesive failure in the cement (C), mixture of adhesive failure at the cement–restorative material interface and cohesive failure (A), failure of the restorative materials (F).

### 2.4 Statistical analysis

The bond strengths were analysed using a statistical software EZR (Saitama Medical Center, Japan). The mean and standard deviation were calculated by using n = 11 raw data for each group. The one-way analysis of variance (ANOVA), followed by Tukey's post-hoc test, was carried out for the multiple comparisons between the groups. The significance level was set at 0.05 for all analyses.

## Declaration of Competing Interest

The authors declare that they have no known competing financial interests or personal relationships which have, or could be perceived to have, influenced the work reported in this article.

## Appendix Supplementary materials

Image, application 1

Image, application 2
